# A Multi-Laboratory, Multi-Platform Analysis of the Multi-Attribute Method

**DOI:** 10.3390/ph18111613

**Published:** 2025-10-25

**Authors:** Joshua Shipman, Mercy Oyugi, Tim Andres Marzan, Ilan Geerlof-Vidavsky, Douglas Kirkpatrick, Hongbin Zhu, Milani Rasangika, Sarah Rogstad

**Affiliations:** 1 Office of Pharmaceutical Quality Research, Center for Drug Evaluation and Research, US Food and Drug Administration, Saint Louis, MO 63110, USA; 2 Office of Product Quality Assessment, Center for Drug Evaluation and Research, US Food and Drug Administration, Silver Spring, MD 20903, USA; 3 BridgeBio, Palo Alto, CA 94304, USA; 4 Office of Science and Engineering Laboratories, Center for Devices and Radiological Health, US Food and Drug Administration, Silver Spring, MD 20903, USA

**Keywords:** mass spectrometry, multi-attribute method, quality control, monoclonal antibodies

## Abstract

**Background/Objectives**: The multi-attribute method (MAM) has found diverse use in the analytical characterization of therapeutic protein products during their development and production. As the MAM matures it has the potential to enter quality control (QC) laboratories, consolidating and replacing many less informative chromatographic techniques; however, this requires an appropriate risk assessment and understanding of method capability. **Methods**: A validated MAM approach was used to quantify product quality attributes (PQAs) using three different mass spectrometers across two laboratories; the results were compared to conventional hydrophilic interaction chromatography–fluorescence detection (HILIC-FLD) and cation exchange chromatography–ultraviolet (CEX-UV) techniques. **Results**: Stressed, long-term, and accelerated stability studies were performed, and their effects on glycosylation, deamidation, oxidation and N- and C-termini were quantified. **Conclusions**: Overall, the inter-instrument inter-laboratory data provided here showed important considerations for transferring methods between laboratories and establishing the correlation between the MAM and conventional data, elements which are necessary to transition the MAM to the QC environment and ultimately achieving the goal of replacing orthogonal QC methods.

## 1. Introduction

Therapeutic monoclonal antibodies (mAbs) are complex macromolecules produced through recombinant cell expression whose primary amino acid sequence is heterogeneously modified throughout the product’s lifecycle. Understanding the product quality attributes (PQAs) of a therapeutic protein provides an understanding of product quality risks and aids in the development of control strategies to mitigate those risks. Physicochemical characterization of mAb therapeutics has conventionally relied on a panel of analytical techniques, with each measurement traditionally reporting non-specific ensemble averages of a particular characteristic, for example, the separation of acidic or basic species separated by cation-exchange chromatography (CEX-UV).

The multi-attribute method (MAM) leverages the specificity and sensitivity of mass spectrometry (MS) to quantify PQAs at the peptide level, potentially serving as a replacement for conventional techniques and providing a more direct measurement of individual PQAs. Typically, the MAM uses conventional peptide mapping procedures followed by liquid chromatography (LC)-MS analysis to evaluate PQAs at a site-specific level. Variations in sample preparation, such as middle-down digestion, and instrumentation, such as capillary zone electrophoresis (CZE-MS) and ion mobility (IM)-MS, have also been reported [1,2,3,4]. The MAM framework has also been applied beyond traditional monoclonal antibodies to other products such as fusion proteins, antibody–drug conjugates (ADCs), and erythropoietin (EPO) [5,6,7].

The MAM has been applied throughout the biopharmaceutical pipeline during candidate selection, process development, and manufacturing. During developmental stages, the MAM has been used to monitor glycosylation, C-terminal lysine clipping, N-terminal cyclization, oxidation, deamidation, isomerization, and sequence variants [8,9,10,11,12]. Recently, the MAM has been introduced into the quality control (QC) environment to monitor oxidation and deamidation [13]. A review of Biologics License Applications (BLAs) found five instances of MS usage for QC in the period from 2016 to 2020, while MS had not been used in this capacity in the period from 2000 to 2016 [14,15]. As the MAM transitions to an established QC technique, instrument vendors have begun offering automated sample preparation and data analysis workflows that intend to increase throughput and allow MAM implementation in laboratories less specialized in LC-MS characterization [6,16,17,18]. When developing the MAM for use during different parts of a therapeutic protein product’s lifecycle, MAM procedures should demonstrate that they are suitable for the intended purpose. When transferring the MAM into the QC lab, the risk associated with the use of this method should be lowered by the product-specific knowledge gained during characterization. Replacing conventional QC methods with the MAM requires risk assessment, control strategies, and continuous monitoring. The change in analytical procedures is established as acceptable through bridging studies, which demonstrate that relevant critical quality attributes (CQAs) are sufficiently captured by showing a strong correlation between methods [13]. The benefits of replacing conventional methods with the MAM include reducing the number of analyses required, reducing the rate of batch failure caused by non-CQA-related changes to products, and the ability to monitor new impurities using new peak detection (NPD) software [19,20,21].

In this study, a previously developed MAM approach was performed at two laboratories using three instruments. The inter-instrument and inter-laboratory results compared orbitrap (OT-MAM) and time-of-flight (TOF-MAM) data. Long-term, accelerated, and stressed stability studies were conducted, and MAM-monitored PQAs were compared to conventional chromatographic methods. Widespread regulatory adoption of the MAM relies on the ability to correlate results with those from conventional methods and transfer between environments throughout the biopharmaceutical lifecycle. The results herein provide valuable technical insights as the method is being evaluated for adoption as a QC tool by the biopharmaceutical industry.

## 2. Results

### 2.1. Overview and System Comparison

The MAM approach used herein was previously described for the monitoring of 21 rituximab PQAs [21]. In the current study, samples were subjected to stressed, long-term, and accelerated stability testing; sample preparation for stability testing was conducted at Site 2 and shipped to Site 1. A summary of the sample conditions and the attributes monitored are described in Table 1. Three MS datasets were collected across two laboratories. Two models of orbitraps (OT1 and OT2) were used along with a Q-TOF (TOF1). Sample digestion and clean-up was performed on-site at each laboratory: OT1 and TOF1 samples were prepared at Site 1, while OT2 samples were prepared at Site 2.

System suitability for all three instruments was qualified by injecting PRTC, a synthetic peptide mixture, every 11 or 12 injections. Retention time, peak area fractional abundance, and mass error variance were measured on all three systems. The system suitability results can be found in Tables S5 and S6 and Figure S1. Retention times were more consistent between OT1 and TOF1, likely because experiments were performed using the same HPLC column. All instruments met the system suitability requirement for retention time of CV < 5.0%. Fractional abundance of peak areas varied between the datasets, but all systems met the system suitability requirement of CV% < 15.0%. These variations may be explained by differences in source conditions between instruments. Transfer between systems required an understanding of instrument capabilities. For example, the mass error system suitability was raised from 5 PPM to 10 PPM for the TOF which is in line with the instrument specifications. One potential aspect of system suitability that is not addressed in this study is variation in sample preparation. To incorporate sample preparation into system suitability testing, a protein standard could be digested and analyzed instead of using a peptide mixture, which only assesses system suitability of the instrumental analysis.

Full method validation for the MAM used in this study was performed on OT2 in accordance with the International Council for Harmonisation’s (ICH) guidelines for the validation of analytical procedures [21,22]. ICH Q14 guidelines for analytical procedure development includes the transfer of methods to a new environment [23]. In accordance with ICH guidelines, the suitability of the analyses on OT1 and TOF1 was demonstrated by showing that the results were comparable to OT2 as discussed for individual PQAs in Section 2.2, Section 2.3, Section 2.4 and Section 2.5; transfer within a regulatory environment would require that differences are evaluated for their potential impact on product specifications.

The transfer of the analysis to the TOF1 system required additional evaluation due to the system’s decreased resolution. High-resolution instruments are important for method development and product characterization; however, it is typical for methods to be transferred to robust, lower-cost platforms for QC use [24,25]. The resolution of OT systems used was 140,000, while the TOF system had a resolution of >10,000 at full width at half maximum (FWHM). System suitability for the lower-resolution instrument was demonstrated by evaluating the analytes with the highest resolution requirements; peptide glycosylation at N301 resulted in the highest mass analytes and therefore required the highest MS resolution. These peptides were isotopically resolved on TOF1, which demonstrated that the lower-resolution instrument was still fit for purpose.

### 2.2. N-Linked Glycans

**Figure 1:**
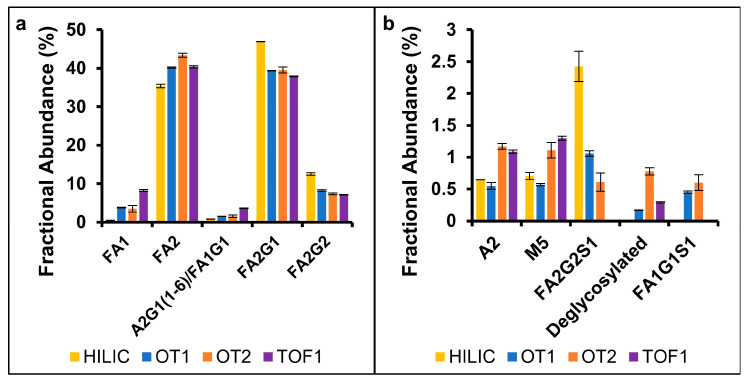
Comparison between HILIC-FLD released 2-AB derivatized glycan quantification and MAM glycopeptide quantification on three MS instruments for high-abundance (**a**) and low-abundance (**b**) species.

N-linked glycans are oligosaccharides attached to the peptide backbone at an asparagine residue. The MAM results were compared to released glycans derivatized with 2-AB and separated with HILIC-FLD, the standard method for mAb glycan profiling. The results are shown in Figure 1. HILIC-FLD provided separation for isobaric branched glycoforms, which was not achieved by the MAM due to a lack of glycopeptide selectivity. Comparison of the three MS datasets found that the average CVs for OT1 (3%) and TOF1 (2%) were lower than OT2 (12%), and four glycoforms quantified on OT2 were found to have particularly high CVs (greater than 20%), while the CVs of other OT2 glycoforms were comparable to the other systems. Differences between source settings and ESI in-source fragmentation may contribute to variance between systems [26,27].

Two glycoforms (FA1G1S1, de-glycosylated) were exclusively detected by the MS methods. The A2G1(1-6) and FA1G1 glycans likely co-eluted using HILIC-FLD as they are known to have similar retention times, and the MAM results indicated that FA1G1 was the higher abundance glycoform, which was consistent with other reports of rituximab glycosylation [28,29]. The FA2G2S1 and FA1G1S1 glycoforms were not detected by TOF analysis; these glycoforms were low-abundance and may not be detected due to lower system sensitivity. In regulatory applications, the potential impact on product specifications would need to be assessed, and sensitivity would need to be sufficient to quantify relevant PQAs. Galactosylated glycans were found at a higher abundance using FLD relative to MS, which was consistent with previously published results and likely due to the differences in ionization and in-source fragmentation of the glycoforms [27,30]. In QC environments, comparison to a reference standard with historical data may be used to evaluate and control the effect of system variance [26].

The fractional abundance of glycoforms quantified by HILIC-FLD and the MAM can be directly correlated, and it is well-established that the two methods can generate comparable results [9,10,31,32]. However, each method was limited in the capability to characterize certain glycoforms. For example, only FLD was able to quantify glycoform branching information, while the MAM provided information about vacant glycosylation sites and may offer additional data for products with additional glycosylation sites, glycation-related PQAs, or O-linked glycosylation [32,33]. Intact protein chromatography can provide glycoform-specific information for correlation, but these methods generally identify fewer species than glycan and glycopeptide analysis [34,35]. A recent study demonstrated that glycan-related MAM data for EPO could be correlated to capillary zone electrophoresis (CZE) and high-performance anion exchange chromatography combined with pulsed amperometric detection (HPAEC-PAD). These results provided good examples of how MAM data can be correlated to glycosylation macro-heterogeneity along with typical glycoform-based analysis [5].

### 2.3. Deamidation

**Figure 2:**
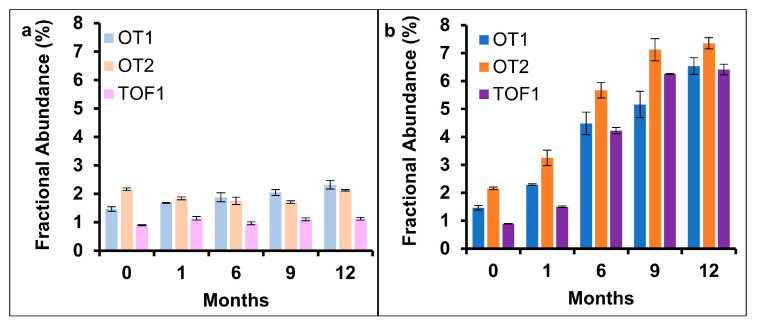
Stability study results of N388 deamidation quantified by MAM. Under expected storage conditions (5 °C), (**a**) deamidation does not substantially increase, while under elevated temperature (25 °C, 60% R.H.), (**b**) deamidation increases with product age.

**Figure 3:**
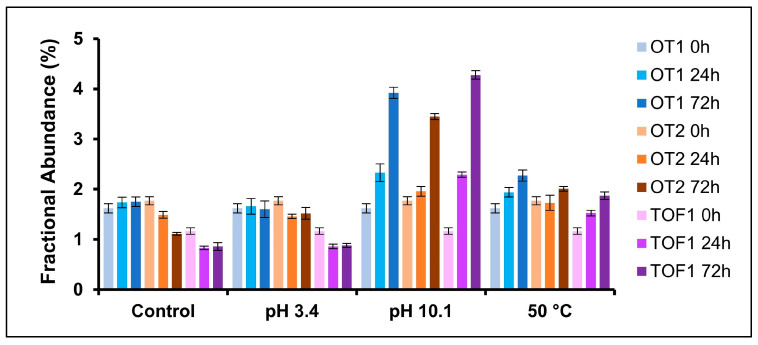
Stressed stability study results of N388 deamidation quantified by MAM. Samples subjected to pH and temperature stressors for 0, 24, or 72 h. Deamidation did not increase for control or acid-stressed groups but increased for base- and temperature-stressed samples.

Protein deamidation can affect protein function and therapeutic efficacy [36]. The validated MAM quantified deamidation at one mAb site, N388. A timepoint stability study was performed and N388 deamidation was evaluated. Samples were tested at 0, 1, 6, 9, and 12 months after storage at both 5 °C (long-term) and 25 °C/60% relative humidity (RH) (accelerated) (Figure 2). Deamidation did not detectably increase for the 5 °C sample set. A linear increase (R^2^ > 0.9) was found for all three datasets when samples were stored at 25 °C/60% RH, with a rate of increase varying between 0.40 and 0.50% per month. Deamidation was also monitored for mAbs subjected to short-term pH and temperature stressors (Figure 3). Control and pH 3.4 samples did not show a considerable increase in N388 deamidation across the timepoints sampled. pH 10.0 and 50 °C stressed samples showed a linear increase in N388 deamidation, with pH 10.0 having the greater rate of increase.

**Figure 4:**
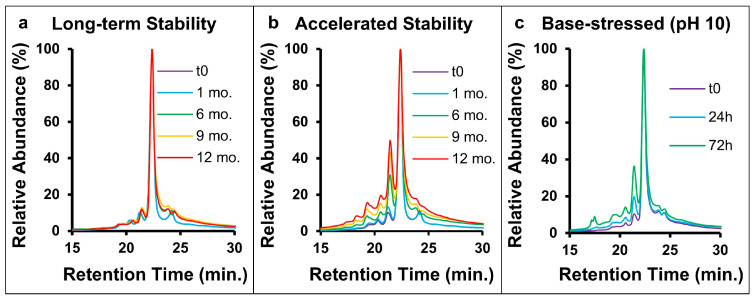
CEX-UV results for (**a**) long-term stability, (**b**) accelerated stability, and (**c**) base-stressed samples. Chromatograms for long-term stability samples were consistent while an increase in acidic (earlier eluting) variants was observed for accelerated stability and base-stressed samples.

CEX was selected as a comparative method. Representative chromatograms of CEX stability data are shown in Figure 4a–c. Four acidic peaks were identified and monitored; long-term stability samples had no quantifiable increase in acidic variants, while three peaks in the accelerated stability samples showed a linear increase (R^2^ > 0.9), with deamidation increasing at a rate of 0.13–1.5% per month. The base-stressed samples showed an increase in acidic species; however, unlike the stability data, increases were largely driven by a single peak. The rate of increase was higher in the base-stressed samples (0.19% per hour) compared to the accelerated stability samples (0.04% per hour), which aligns with MAM data. No notable increase in acidic variants was found in the acid-stressed or control samples.

**Figure 5:**
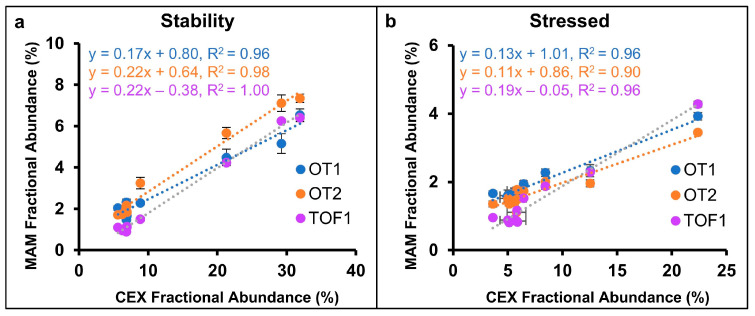
Correlation between N388 deamidation quantified by MAM and the sum of acidic peaks quantified by CEX-UV for (**a**) long-term and accelerated stability and (**b**) stressed studies.

The sum of acidic CEX peaks were plotted against N388 deamidation quantified by the MAM in Figure 5a,b to demonstrate the correlation between the methods. Linearity was observed (R^2^ > 0.9) for the three datasets using both stressed and stability data. Slopes ranged from 0.11 to 0.22, which is logical given that CEX peak area represented the total of multiple acidic species, while MAM data represented a specific deamidation event. Ideally, in a product development workflow, individual CEX peaks would be isolated through fractionation or multi-dimensional LC to identify specific PQAs, which could then be directly correlated [37,38,39]. The specificity of the MAM is advantageous as deamidation sites known to effect bioactivity can be individually monitored [13]. Other conventional characterization techniques that can provide orthogonal information include anion-exchange (AEX) and hydrophobic interaction chromatography (HIC). Pre-peaks and post-peaks can be directly compared to MAM data following fractionation and confirmation of contributing species, and indirect correlations can also be made [9].

### 2.4. Oxidation

**Figure 6:**
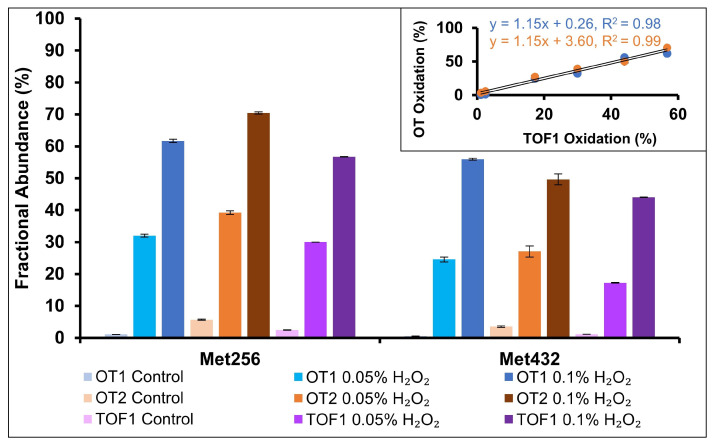
MAM quantification of M256 and M432 oxidation in H_2_O_2_-stressed samples. Increased oxidation was observed across the three MS instruments.

mAb oxidation is a CQA which can potentially lead to a loss of structure and function; residue-specific methionine oxidation can impact antibody binding, highlighting the importance of site-specific quantification [19,40,41]. Samples in this study were stressed with 0.05% and 0.1% hydrogen peroxide for 5 h. Two methionine residues, M256 and M432, were identified as susceptible to oxidation. M254 and M432 are conserved residues in the Fc region of IgG_1_ antibodies and are known to be surface exposed and susceptible to oxidative stress [42]. The MAM results for the oxidation of the two peptides are shown in Figure 6. Oxidation increased with the concentration of hydrogen peroxide and was comparable between the three instruments as plotting the two OT datasets against the TOF data found nearly identical slopes and R^2^ > 0.98 (Figure 6 inset).

**Figure 7:**
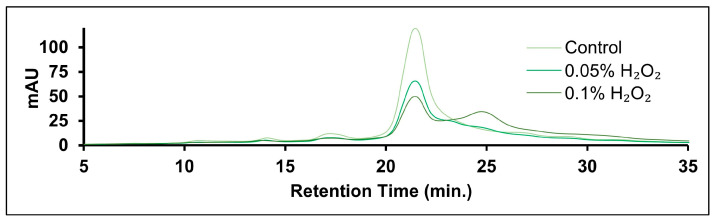
CEX-UV results for H_2_O_2_-stressed samples. An increase in basic (later-eluting) species could be observed but were poorly resolved from the primary mAb species.

CEX was selected as a comparative method for oxidation. An example chromatogram of hydrogen peroxide stressed samples is shown in Figure 7. While the chromatogram varied with increased oxidative stress, the low resolution between peaks prevented quantitative analysis. Results for Fab oxidation quantified by the MAM have been successfully correlated to HIC results [19]. AEX may also provide clearer results for oxidation-stressed samples. However, the high isoelectric point of rituximab (9.4) likely precludes the mAb from being analyzed by AEX [43,44].

### 2.5. C- and N-Terminal Variants

**Figure 8:**
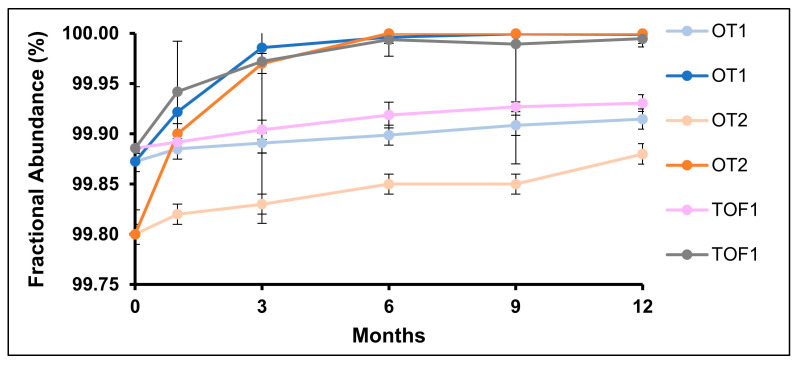
Stability study results of heavy-chain Q1 pyro-Q formation quantified by MAM. Under long-term storage conditions (5 °C, light data points), pyro-Q increases linearly in the three MS datasets over 12 months, while under accelerated stability conditions (25 °C/60% R.H., dark data points), complete pyro-Q formation is observed after six months.

The N-termini of IgG_1_ mAbs contain conserved glutamic acid residues which can be converted to pyroglutamic acid (pyro-Q), lowering mAb molecular weight and isolectric point. The results for the stability of pyro-Q formation are shown in Figure 8. Long-term stability samples showed a linear increase across instruments over twelve months (R^2^ > 0.9), while accelerated stability samples increased more rapidly and reached >99.99% occupancy when tested at 6 months. The observed increase in pyro-Q in the MAM could not be correlated to CEX-UV data. Correlations could not be made because CEX-UV lacks specificity and sensitivity; stability and stressed studies pre-dominantly detected an increase in acidic variants, which masked the relatively small increase of pyro-Q.

**Figure 9:**
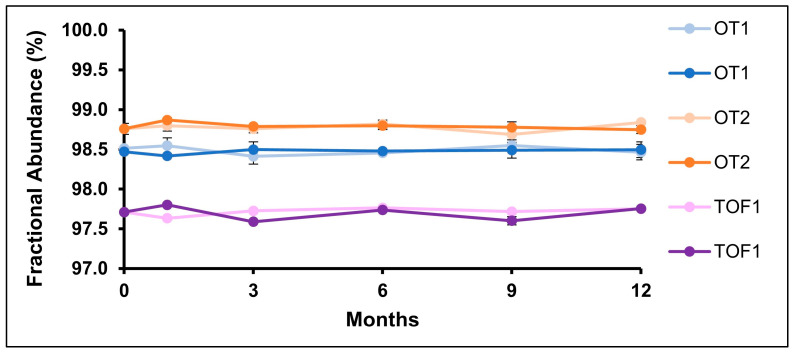
Stability study results of heavy-chain K451 clipping quantified by MAM. K451 clipping was consistent for long-term (5 °C, light data points) and accelerated stability (25 °C/60% R.H., dark data points) samples for all three MS datasets.

The C-terminus of the heavy chain contains a lysine residue which is clipped during protein processing. C-terminal lysine incorporation is known to be sensitive to the manufacturing process, and monitoring of this attribute can ensure lot-to-lot consistency [45]. The clipped form of the C-terminal heavy chain peptide was quantified (Figure 9). Stability testing indicated that lysine clipping was consistent across both temperature conditions for all three instruments, with minor differences in the quantified amount. Studies have shown that results for the MAM and reduced capillary electrophoresis sodium dodecyl sulfate (rCE-SDS) or capillary gel electrophoresis (CGE) can be correlated for clipped species. However, conventional methods may lack the necessary sensitivity to detect the small amount of clipping present in rituximab [10,46]. Basic fractions of ion exchange chromatography (IEC) separations have also been correlated to the MAM and size-exclusion chromatography-UV (SEC-UV) in method-bridging studies, which were used to remove IEC from a marketing application [13].

## 3. Discussion

The MAM offers comprehensive analysis of therapeutic proteins at the molecular level and provides NPD capabilities. The data in this report shows that MAM/NPD studies provide higher sensitivity, resolution, and overall higher-information-content data compared to traditional chromatographic data used for biomanufacturing control strategies. Thus, the MAM improves the capability to identify product changes and, potentially, to control batch to batch consistency of biologic products. The biopharmaceutical industry has embraced the MAM at early stages of product development. Many publications reference use of the MAM workflow, and an informational general chapter on the topic was recently released by the United States Pharmacopeia [47]. However, despite this increase in available information, regulatory submissions for routine use of the MAM in QC settings are still limited. The major challenges associated with implementation of the MAM in regulatory submissions for routine QC testing include correlating data between methods and validating method transfer between laboratories. The studies conducted herein serve as a platform to discuss these challenges.

Transferring a developed MAM procedure to a different testing environment requires a thorough evaluation of changes to ensure the procedure remains suitable; method parameters throughout the analytical workflow should be considered [47]. In most instances, transferring the MAM between laboratories would not change the principles of the analytical procedure, which should decrease the risk associated with the change [23]. In this study, a method that was previously developed and validated on an OT instrument was transferred to a lower-resolution TOF instrument. However, in the TOF workflow the monitored attributes were still isotopically resolved, which was comparable to the OT results and in line with instrument specifications. In instances where a method is transferred to a lower-resolution instrument, the extent of change in the analytical procedure would be increased, and suitability may need to be demonstrated further [25]. The validated method used herein used an ultra-fast digestion procedure. Results for other rapid peptide mapping digest methods have demonstrated differences between vendor-specific digestion enzymes [48]. Variance in sample preparation could also cause artifactual increases in deamidation or oxidation. Ionization conditions can be difficult to reproduce across instruments and can impact measurements of labile glycosidic bonds; the potential impact of these changes on the defined specifications may be evaluated and found to be low as repeatable glycosylation profiles and the absence of potentially immunogenic glycans may still be demonstrated.

A major roadblock for widespread MAM adaptation is the establishment of a strong correlation between the MAM and comparator methods. Ideally, a correlation between a validated MAM procedure and a conventional method would be directly established for a specific analyte; however, offsets between conventional and MAM data are to be expected [47]. In some instances (e.g., individual glycoforms), correlation can be relatively straightforward, while in others (e.g., identifying a charge-variant component in a CEX peak containing multiple co-eluting components), additional sample analysis may be required. When it is not feasible to establish direct correlation, indirect correlation could be considered, such as looking at the sum of acidic or basic species. The requirements for method correlation differ depending on a product’s stage in the regulatory lifecycle. Approved BLAs require extensive bridging studies to ensure that the replacement of approved methods does not leave gaps in the product quality testing. In these cases, a full validation of the MAM and a comparative analysis of representative samples would be recommended because the principle analytical procedures differ [23]. Products still under investigational new drug (IND) status offer more flexibility for MAM implementation because the MAM can be optimized concurrently with product development, and product specifications can be defined using the MAM. Some conventional analytical methods may be determined to be unnecessary if the MAM is demonstrated to fully capture all necessary PQAs of the product profile based on a thorough risk assessment. Due to the increased requirements needed to replace conventional methods at the BLA stage, increased use of the MAM in future submissions is more likely to occur earlier in the product lifecycle at the IND stage.

Data generated at different stages of a product’s lifecycle can be adapted to correlate the MAM and conventional methods. In this study, samples were subjected to chemical and physical stress conditions and long-term and accelerated stability conditions. PQAs sensitive to those conditions were measured and compared using the MAM, HILIC-FLD, and CEX. Stability testing occurs during product development, and correlating the MAM and other methods at this stage can be carried out while concurrently determining specification limits [47,49]. During product development, correlation can also be established by using a mixture of standards with known compositions. For some analytes (e.g., clipping variants), these mixtures can be used to establish linearity over a range of values that are difficult to establish using the product itself [46]. Large historical multi-batch datasets could be used to establish the correlation between methods, for example, in a bridging study to remove a QC method from routine analysis. Historical datasets also are useful for demonstrating method correlation at product-relative abundances. The current study was performed using a commercially available antibody with three FDA-approved biosimilars, and the methods herein could also be applied to lot analysis to identify product specific differences.

Ultimately, the successful transfer of the MAM into regulatory submissions and routine QC testing will enhance product understanding and improve product control strategies, thus ensuring the quality of therapeutic protein drug products. While challenges remain in navigating regulatory requirements and standardizing methods to demonstrate the correlation to conventional methods, the MAM has been embraced as a versatile platform technology for mAb characterization, and as the approach matures, increased use of the MAM throughout the biopharmaceutical lifecycle is anticipated.

## 4. Materials and Methods

### 4.1. Materials

One lot of rituximab (10 mg/mL) manufactured by Genentech was purchased for analysis. Pierce™ Retention Time Calibration (PRTC) Mixture, dithiothreitol (DTT), iodoacetic acid (IAA), Tris-HCl, Pierce™ trypsin protease, Zeba™ spin desalting columns (7 K MWCO, 0.5 mL), formic acid (FA), Optima LC/MS-grade acetonitrile (ACN), and Optima LC/MS-grade water were purchased from Thermo Fisher Scientific (Waltham, MA, USA). Guanidinium hydrochloride (GdnHCl) and ammonium bicarbonate (NH_4_HCO_3_) were purchased from Sigma-Aldrich (St. Louis, MO, USA). 

### 4.2. MAM Peptide Mapping with LC-MS

Samples were digested as previously reported [21]. Detailed sample preparation and additional procedures for stressed, long-term, and accelerated stability samples can be found in the Supporting Information.

Data were collected on three mass spectrometers: a Thermo Scientific Q Exactive HF-X (OT1; Waltham, MA, USA), Thermo Scientific Q Exactive (OT2; Waltham, MA, USA), and a Waters Bioaccord (TOF1; Milford, MA, USA). LC separation was performed using either a Thermo Vanquish (OT1; Waltham, MA, USA), Thermo Accela (OT2; Waltham, MA, USA), or Waters Acquity H-Class (TOF1; Milford, MA, USA) system and an Agilent Zorbax C18 300 SB reversed phase column (300 Å, 1.8 µm, 2.1 mm × 150 mm, Santa Clara, CA, USA). Column temperature was 50 °C, and flow rate was 0.250 mL/min. Mobile Phase A (MPA) was 0.1% FA in water and Mobile Phase B (MPB) was 0.1% FA in acetonitrile. For detailed methods see the Supporting Information.

Samples were injected in triplicate in a randomized injection sequence. 10 µL of sample was injected, and blank and system suitability injections were bracketed around every 11 or 12 test samples.

Fractional abundances of identified PQAs were quantified using workbooks developed using Chromeleon, Thermo Fisher Scientific, version 7.2 SP5 (OT1), Chromeleon, Thermo Fisher Scientific, version 7.2 SP5 (OT2) or the Waters peptide MAM application, version 1.4. (TOF1).

### 4.3. Hydrophilic Interaction Chromatography–Fluorescence Detection (HILIC-FLD)

mAb glycans were released and derivatized using 2-aminobenzamide (2-AB). HPLC separation was performed using an Agilent 1290 system (Santa Clara, CA, USA) and a Waters Acquity BEH glycan column (2.1 mm× 150 mm, 1.7 µm, Milford, MA, USA). MPA was 10 mM ammonium formate, pH 4.5, and MPB was acetonitrile. For detailed methods see the Supporting Information.

### 4.4. Cation Exchange Chromatography–Ultraviolet Detection (CEX-UV)

Charge variant analysis was performed using CEX-UV. HPLC separation was performed using an Agilent 1290 system and an Agilent BioMab, NP5 column (2.1 mm× 250 mm, 5 µm, Santa Clara, CA, USA). MPA was 10 mM phosphate, pH 7.76, and MPB was 10 mM phosphate with 100 mM NaCl. For detailed methods see the Supporting Information.

## Figures and Tables

**Table 1:** Summary of rituximab stability conditions in this study.

Condition	Samples	Site Monitored (Heavy Chain)
Long-term stability, 5 °C	0, 1, 6, 9, 12 months	N388, N301 glycans, Q1, K451
Accelerated stability, 25 °C/60% RH	0, 1, 6, 9, 12 months	N388, N301 glycans, Q1, K451
Oxidative stress	0%, 0.05%, 0.1% H_2_O_2_	M256, M432
pH 3.4	0, 24, 72 h	N388
pH 10.0	0, 24, 72 h	N388
50 °C	0, 24, 72 h	N388

## Data Availability

The original contributions presented in this study are included in the article/Supplementary Material. Further inquiries can be directed to the corresponding author.

## Supplementary Materials

The following supporting information can be downloaded at https://www.mdpi.com/article/10.3390/ph18111613/s1, Detailed experimental protocols, Table S1: HLIC-FLD Parameters, Table S2: HILIC-FLD HPLC gradient, Table S3: CEX-UV HPLC parameters, Table S4: CEX-UV HPLC gradient, Table S5: Retention time (RT) data for PRTC system suitability standard, Table S6: Mass error (PPM) and fractional abundance (FA) data for PRTC system suitability standard, Figure S1: Fractional abundance and retention times of PRTC system suitability standards.

## Abbreviations

The following abbreviations are used in this manuscript.

2-AB	2-aminobenzamide
ACN	Acetonitrile
ADC	Antibody–drug conjugate
AEX	Anion exchange chromatography
BLA	Biologics License Application
CEX	Cation exchange chromatography
CGE	Capillary gel electrophoresis
CQA	Critical quality attributes
CZE	Capillary zone electrophoresis
DTT	Dithiothreitol
EPO	Erythropoietin
ESI	Electrospray ionization
FA	Formic acid
FLD	Fluorescence detection
FWHM	Full width at half maximum
GdnHCl	Guanidinium hydrochloride
HIC	Hydrophobic interaction chromatography
HILIC	Hydrophilic interaction chromatography
HPAEC-PAD	High-performance anion exchange chromatography combined with pulsed amperometric detection
HPLC	High-performance liquid chromatography
IAA	Iodoacetic acid
ICH	International Council for Harmonisation
IEC	Ion exchange chromatography
IgG1	Immunoglobulin G1
IM	Ion mobility
IND	Investigational new drug
LC	Liquid chromatography
mAb	Monoclonal antibody
MAM	Multi-attribute method
MPA	Mobile Phase A
MPB	Mobile Phase B
MS	Mass spectrometry
MWCO	Molecular weight cut-off
NH_4_HCO_3_	Ammonium bicarbonate
NPD	New peak detection
OT	Orbitrap
PQA	Product quality attributes
PRTC	Pierce Retention Time Calibration
pyro-Q	Pyroglutamic acid
QC	Quality control
rCE-SDS	Reduced capillary electrophoresis sodium dodecyl sulfate
RH	Relative humidity
RT	Retention time
SEC	Size-exclusion chromatography
TOF	Time-of-flight
UV	Ultraviolet
