# Supplementary material for: A Multi-Laboratory, Multi-Platform Analysis of the Multi-Attribute Method
Source: Pharmaceuticals (Basel). 2025 Oct 25;18(11):1613. doi: 10.3390/ph18111613 (PMC12655225; doi:10.3390/ph18111613)

Oxidation Stressed Samples

Rituximab samples were treated with 0.05% and 0.1% H_2O_2. For the 0.05% H_2O_2, 1 µL of 10% H_2O_2 was added to 199 µL rituximab for a total volume of 200 µL and a final rituximab concentration of 9.95 mg/mL. For the 0.1% H_2O_2, 2 µL of 10% H_2O_2 was added to 198 µL rituximab for a total volume of 200 µL and a final rituximab concentration of 9.90 mg/mL. The samples were then incubated for 5 hours at 2-8 °C along with 200 µL of control samples without any H_2O_2. The samples were then buffer exchanged into the rituximab formulation buffer to remove the H_2O_2. The samples were then aliquoted for MAM analysis and stored at -80 °C until needed.

pH Stressed Samples

The rituximab samples were stressed at pH 3.4 and pH 10.0, using buffers with base ingredients and concentrations similar to the formulation buffer (0.7 mg/mL PS80, 9 mg/mL NaCl, 7.35 mg/mL sodium citrate dihydrate at pH 6.5). The pH 3.4 buffer contained 0.7 mg/mL PS80, 9 mg/mL NaCl, and 7.35 mg/mL sodium citrate dihydrate, while the pH 10.0 buffer contained 0.7 mg/mL PS80, 9 mg/mL NaCl, and 1.97 mg/mL ammonium bicarbonate. For each condition, two 200 µL samples were buffer exchanged into the appropriate pH buffer, followed by incubation of both samples at RT, one for 24 hours and the other for 72 hours. For each incubation duration, non-buffer exchanged controls were also incubated along with the samples. At the end of each incubation period, the pH-treated samples were buffer exchanged into the rituximab formulation buffer. The samples were then aliquoted for MAM analysis and stored at -80 °C until needed.

Thermally Stressed Samples

Two 200 µL samples were incubated at 50 °C, one for 24 hours and the other for 72 hours. At the end of each incubation period, the samples were aliquoted for MAM analysis and stored at -80 °C until needed. Since the pH and 50 °C samples were stressed concurrently and had similar incubation times, the pH control samples were used as controls for the 50 °C stress as well.

Stability Samples

200 µL was aliquoted for each sample. Samples were stored at 5 °C (long-term stability) or 25 °C/60% relative humidity (RH)(accelerated stability) for 1, 6, 9 or 12 months). The time 0 samples were obtained directly from the -80 °C freezer without being subjected to stability conditions.

MAM Sample Preparation

1. 5 µL of 10 mg/mL Rituximab was diluted to 1 mg/mL in a denaturing buffer containing 7.5 M GdnHCl, 250 mM Tris, pH 8.3.
2. 1 µL 500 mM DTT was added and samples were incubated for 30 minutes at room temperature.
3. 2 µL 500 mM iodoacetic acid was added and samples were incubated for 20 minutes at room temperature in the dark.
4. Reaction was quenched with 1 µL 500 mM DTT.
5. Samples were de-salted and buffer exchanged into the digestion buffer (100 mM ammonium bicarbonate, pH 8.0), using Zeba spin desalting columns as follows:
    1. Columns were centrifuged at 1500g for one minute, flow-through was discarded.
    2. Column was washed with 300 µL digestion buffer then centrifuged at 1500g for one minute, flow-through was discarded.
    3. Step (2) repeated two additional times.
    4. Washed column was transferred to clean Eppendorf tube
    5. Sample loaded on top of resin, 15 µL digestion buffer added on top of resin bed
    6. Centrifuge at 1500g for two minutes, flow-through contains sample
6. 5 µL 1 mg/mL trypsin added to samples (1:10 enzyme:substrate ratio), samples incubated for 30 minutes at 37°C.
7. 3 µL formic acid added to samples to quench digest

Analysis of 2-AB derivatized glycans with HILIC-FLD

Table S1: HILIC-FLD HPLC Parameters

**Parameter**	**Setting**
Flow Rate	0.5 mL/min
Injection Volume	10.0 µL
Column Temperature	60 ºC
Auto-sampler Temperature	15 ºC
Detector	FLD
Excitation λ	330 nm
Emission λ	420 nm
Run Time	47.5 minutes

Table S2: HILIC-FLD HPLC Gradient

**Time (min)**	**% A**	**% B**	**Flow (mL/min)**
0	25	75	0.50
31.5	35.2	64.8	0.50
32.0	100	0	0.25
35.0	100	0	0.25
36.0	25	75	0.25
40.0	25	75	0.25
41.0	25	75	0.50
47.5	25	75	0.50

Preparation of Samples

Centrifugal Filter Preparation

1. Equilibrate the filter by adding 100 µL of digestion buffer.
2. Add 20 µL of rituximab sample (equivalent to 200 µg).
3. Centrifuge for 5 minutes at 14000 x G.
4. Wash the filter 2x with 350 µL of digestion buffer. Centrifuge for 5 minutes at 14000 x G after each wash.
5. Discard the flow through.

De-Glycosylation

6. Add 48 µL of digestion buffer + 2 µL of PNGase F.
7. Incubate in heating block at 45 °C for 1 hour.
8. Cool down to room temperature.
9. Centrifuge for 5 minutes at 14000 x G.
10. Transfer flow through (≈ 70 ± 10 µL) to a 1.5 mL centrifuge tube.
11. Add 5 µL of 200 mmol/L acetic acid. Vortex.
12. Incubate for 15 minutes at 45 °C.
13. Cool down to room temperature.

Labeling

14. Add 10 µL of labeling reagent (5 µL of 2-AB solution + 5 µL of Reductant solution).
15. Vortex. Centrifuge briefly.
16. Incubate for 2 hours at 65 °C.
17. Cool down to room temperature. Centrifuge briefly.

Removal of excess labeling reagent

18. Rinse required number of cartridges with 1 mL of water and 1 mL of 96% acetonitrile.
19. Add 1 mL of 96% acetonitrile to labeled glycan. Mix.
20. Apply each labeled glycan mixture to individual cartridges.
21. Let sample flow through by gravity.
22. Wash cartridges 3x with 0.75 mL of 96% acetonitrile using low vacuum.
23. Remove from base plate and transfer to new centrifuge tube.
24. Add 100 µL of 20% acetonitrile and allow to absorb into the cartridge.
25. Elute the labeled glycans by gravity, centrifuging briefly if necessary.
26. Mix samples 1:1 with 96% acetonitrile prior to hplc analysis.

CEX-UV Analysis

Table S3: CEX-UV HPLC Parameters

**Parameter**	**Setting**
Flow Rate	200 µL/min
Injection Volume	1.0 µL
Column Temperature	30 ºC
Detector Wavelength	214 nm
Run Time	70 minutes

Table S4: CEX-UV HPLC Gradient

**Time (min)**	**% B**
0	5
36	70
36.5	100
46	100
46.5	5
70	5

Table S5: Retention time (RT) data for PRTC system suitability standard

	OT1			OT2			TOF1		
	RT (avg.)	St. Dev.	CV	RT (avg.)	St. Dev.	CV	RT (avg.)	St. Dev.	CV
SSAAPPPPPR	8.5	0.009	0.1	10.1	0.2	1.5	8.0	0.1	0.8
GISNEGQNASIK	8.7	0.009	0.1	10.7	0.2	1.9	8.23	0.03	0.4
HVLTSIGEK	10.0	0.020	0.2	12.1	0.2	2.0	9.4	0.1	0.7
IGDYAGIK	11.6	0.023	0.2	14.3	0.3	2.1	11.0	0.1	0.5
DIPVPKPK	12.4	0.050	0.4	14.3	0.3	2.1	11.7	0.1	0.7
TASEFDSAIAQDK	14.3	0.029	0.2	17.2	0.3	1.9	13.6	0.1	0.5
SAAGAFGPELSR	17.0	0.051	0.3	19.5	0.3	1.7	16.2	0.1	0.3
ELGQSGVDTYLQTK	20.2	0.040	0.2	22.6	0.3	1.4	19.3	0.1	0.3
GLILVGGYGTR	27.8	0.028	0.1	29.4	0.3	1.1	26.7	0.1	0.2
SFANQPLEVVYSK	28.9	0.029	0.1	30.6	0.3	1.1	27.84	0.05	0.2
GILFVGSGVSGGEEGAR	29.1	0.029	0.1	30.6	0.3	1.1	27.99	0.04	0.2
LTILEELR	34.0	0.034	0.1	36.2	0.3	0.9	32.9	0.1	0.2
NGFILDGFPR	38.1	0.038	0.1	39.9	0.3	0.8	36.89	0.04	0.1
ELASGLSFPVGFK	41.9	0.042	0.1	42.8	0.3	0.7	40.47	0.04	0.1
LSSEAPALFQFDLK	45.6	0.046	0.1	46.8	0.3	0.7	44.19	0.04	0.1

Table S6 Mass error (PPM) and Fractional Abundance (FA) Data for PRTC System Suitability Standard

	OT1				OT2				TOF1			
	PPM	FA (avg.)	St. Dev.	CV	PPM	FA (avg.)	St. Dev.	CV	PPM	FA (avg.)	St. Dev.	CV
SSAAPPPPPR	-0.05	3.10	0.03	1.0	-1.8	3.2	0.2	5.3	1.9	3.60	0.07	1.9
GISNEGQNASIK	-0.59	4.9	0.2	4.7	-2.2	4.2	0.1	2.9	2.4	4.6	0.1	2.2
HVLTSIGEK	-0.16	6.5	0.2	3.2	-1.4	4.9	0.2	3.4	2.3	6.8	0.2	3.2
IGDYAGIK	-0.05	4.3	0.1	2.5	-2.5	2.8	0.2	6.0	2.2	5.44	0.07	1.4
DIPVPKPK	0.03	2.1	0.1	5.3	-2.5	5.8	0.3	4.5	1.3	4.1	0.2	4.2
TASEFDSAIAQDK	-0.68	4.7	0.1	1.5	-2.8	4.5	0.1	1.6	3.9	3.72	0.06	1.5
SAAGAFGPELSR	-0.22	6.0	0.1	1.7	-1.6	7.2	0.1	2.1	2.6	5.49	0.03	0.5
ELGQSGVDTYLQTK	-0.44	7.0	0.1	1.8	-3.6	8.3	0.4	4.6	4.5	5.75	0.06	1.0
GLILVGGYGTR	-0.36	7.80	0.03	0.4	-0.5	7.5	0.1	1.7	2.7	10.74	0.06	0.5
SFANQPLEVVYSK	-0.71	10.0	0.1	1.2	-3.2	8.8	0.2	2.1	5.5	8.16	0.06	0.7
GILFVGSGVSGGEEGAR	-0.56	8.9	0.1	1.4	-3.8	10.1	0.6	6.4	5.1	8.2	0.1	1.4
LTILEELR	-0.38	9.1	0.3	3.1	-0.1	7.1	0.3	4.3	3.6	11.0	0.1	0.9
NGFILDGFPR	-0.41	7.5	0.1	0.8	-0.8	9.0	0.2	2.4	2.7	7.21	0.07	1.0
ELASGLSFPVGFK	-0.66	9.6	0.1	1.4	-2.3	9.8	0.3	3.3	4.2	8.8	0.1	1.3
LSSEAPALFQFDLK	-0.60	8.5	0.4	4.6	-3.6	6.9	0.7	9.9	5.2	6.3	0.1	2.4

Figure S1: Fractional abundance and retention times of PRTC System Suitability Standards

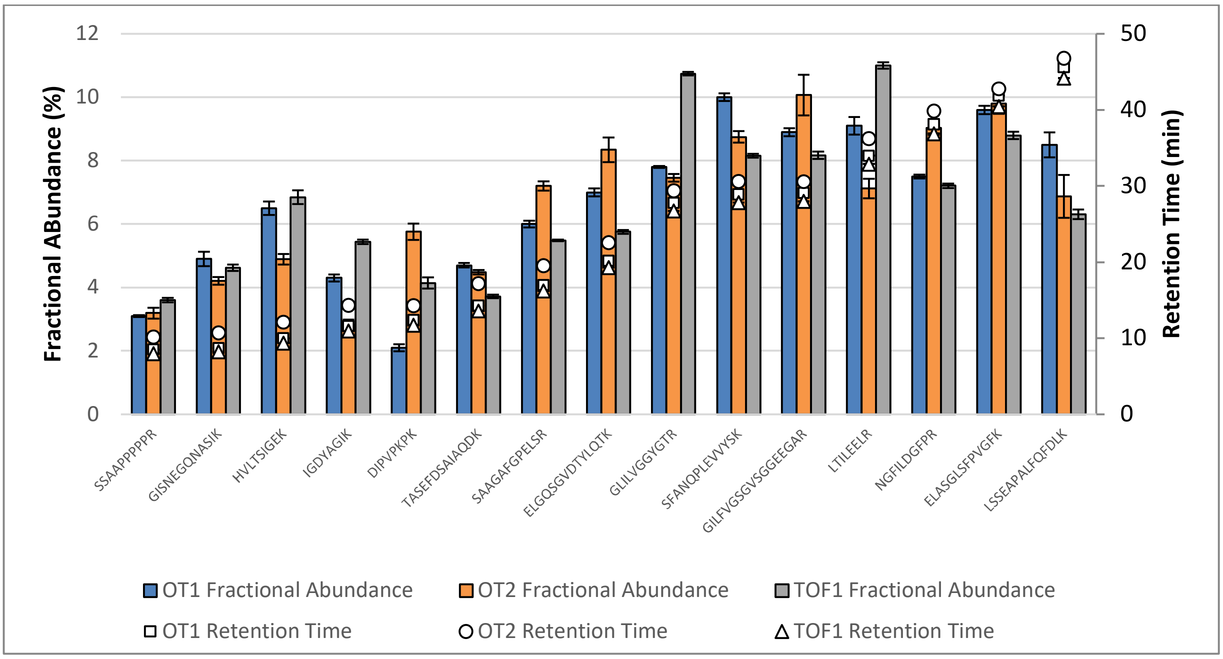

Supplement: Supplementary file 1 [file pharmaceuticals-18-01613-s001.zip › pharmaceuticals-3844153-supplementary.pdf]
